# From empirical regimens to precision prophylaxis: mechanism-based targeting of preeclampsia phenotypes

**DOI:** 10.3389/fphar.2026.1778310

**Published:** 2026-05-12

**Authors:** Chun-Fei Wang, Yu-Fei Zhang, Xue-Feng Jiao, Qiang wei

**Affiliations:** 1 Department of Obstetrics and Gynecology, West China Second University Hospital, Sichuan University, Chengdu, China; 2 Key Laboratory of Birth Defects and Related Diseases of Women and Children, Sichuan University, Ministry of Education, Chengdu, China; 3 Department of Pharmacy/Evidence-Based Pharmacy Center, West China Second University Hospital, Sichuan University, Children’s Medicine Key Laboratory of Sichuan Province, Chengdu, China; 4 NMPA Key Laboratory for Technical Research on Drug Products In Vitro and In Vivo Correlation, Chengdu, China

**Keywords:** aspirin, low-molecular-weight heparin, metformin, preeclampsia, prophylaxis, statins

## Abstract

Currently, prevention strategies for preeclampsia are mainly based on standard low-dose aspirin. However, the clinical efficacy of this regimen exhibits significant heterogeneity across diverse patient populations. This review proposes that future prevention strategies should shift from empirical uniform regimens to individualized precision interventions based on molecular endotypes. Latest evidence indicates that for multiple pregnancies, high-dose aspirin (≥150 mg/d) may be required to neutralize the supraphysiological load of placenta-derived extracellular vesicles. In pregnant women with chronic hypertension, the combination of low-dose aspirin and low molecular weight heparin exert synergistic protection of endothelial glycocalyx integrity, achieving dual benefits of antithrombosis and endothelial protection. For metabolic high-risk populations, statins demonstrate superior therapeutic potential compared to metformin in restoring angiogenic balance by activating the heme oxygenase 1/carbon monoxide pathway and inhibiting the adipokine chemerin. In conclusion, future strategies for preeclampsia prevention should transcend traditional clinical risk stratification to establish mechanism-based precision therapies. This paradigm shift aims to achieve individualized interventions that are matched to the specific molecular endotypes driving disease pathogenesis.

## 1 Introduction

Preeclampsia (PE) is a complex multisystem disorder and one of the most severe, life-threatening complications of pregnancy, affecting 2%–6% of pregnant women worldwide (Yang et al., 2021; Chappell et al., 2021; Roberts, 2024). Earlier onset of PE is associated with more severe disease, leading not only to adverse perinatal outcomes and increasing maternal mortality, but also markedly elevating the maternal long-term risks of cardiovascular disease, diabetes, and dyslipidemia, as well as the higher risk of developing long-term complications such as attention-deficit/hyperactivity disorder in the offspring (Poon et al., 2019; Pittara et al., 2021). At present, the precise diagnoses of PE are still faced with challenges of including several disorders (Roberts, 2024). The only option to arrest the disease is to deliver the fetus and placenta. Meanwhile, therapeutic options for prolonging pregnancy to decrease neonatal morbidity and mortality remain very limited, focusing primarily on symptomatic management such as antihypertensive therapy, prevent seizures, and induce fetal lung maturation (Chappell et al., 2021; Dimitriadis et al., 2023). Therefore, early prediction and prevention are essential for reducing the incidence of PE and its severe complications.

At present, low-dose aspirin (LDA) is the only prophylactic agent broadly endorsed and incorporated into national guidelines. However, its prophylactic efficacy in women at high risk for PE demonstrates variable clinical outcomes across diverse patient cohorts. Specifically, the American College of Obstetricians and Gynecologists (ACOG) recommend a daily dose of 81 mg initiated between 12 and 28 weeks of gestation (optimally before 16 weeks) and continued until delivery, primarily relying on a maternal clinical risk factor checklist (ACOG, 2020). Similarly, the National Institute for Health and Care Excellence advises 75–150 mg daily starting from 12 weeks until birth for high-risk women (NICE, 2019). In contrast, the International Federation of Gynecology and Obstetrics advocates a more integrated first-trimester screening approach, recommending a higher dose of 150 mg to be taken at night, initiated between 11 and 14+6 weeks of gestation and discontinuation at 36 weeks (Poon et al., 2019). This variability explains the inconsistent clinical efficacy of current uniform regimens. Consequently, a transition toward mechanism-based, individualized precision interventions is essential.

Meanwhile, ongoing research into the mechanisms of PE has prompted investigation into whether low-molecular-weight heparin (LMWH), metformin, and statins might also be useful for prevention (Chappell et al., 2021; Dimitriadis et al., 2023). This review integrates current pharmacological mechanisms and pathophysiological insights with existing clinical evidence to systematically evaluate the preventive efficacy of LDA in high-risk pregnancies. Furthermore, it elucidates the therapeutic potential and clinical utility of LMWH, statins, and metformin within the context of precision and individualized preventive strategies.

## 2 Pathophysiology of preeclampsia

The pathogenesis of PE is initiated by impaired trophoblast invasion and inadequate spiral artery remodeling during early placentation, resulting in placental hypoperfusion and focal hypoxia. This hypoxic environment triggers an excessive release of anti-angiogenic factors, predominantly soluble fms-like tyrosine kinase-1 (sFlt-1) and soluble endoglin (sEng), into the maternal circulation. Mechanistically, these mediators promote systemic endothelial dysfunction through two distinct mechanisms: by antagonizing the vasoprotective and pro-angiogenic signaling of vascular endothelial growth factor (VEGF) and placental growth factor (PlGF), and by directly compromising the structural and functional integrity of the endothelial glycocalyx (Ives et al., 2020; Dimitriadis et al., 2023). Specifically, elevated sFlt-1 induces glycocalyx degradation and activates matrix metalloproteinases, thereby driving a vicious cycle of endothelial barrier disruption and vascular hyperpermeability (Atallah et al., 2025). This extensive endothelial injury subsequently disrupts vasomotor homeostasis, characterized by an upregulation of vasoconstrictors (such as endothelin-1) and a downregulation of vasodilators (such as nitric oxide and prostacyclin), ultimately triggering systemic inflammation and microthrombosis. Finally, this pathogenic cascade culminates in the hallmark clinical manifestations of hypertension and proteinuria, accompanied by multi-organ dysfunction involving the renal, hepatic, and neurological systems (Dimitriadis et al., 2023; Ives et al., 2020; Atallah et al., 2025).

However, the upstream triggers of this pathogenic cascade are highly heterogeneous. Clinically, PE has traditionally been classified by gestational age at onset, often conceptualized as ‘placental’ early-onset (<34 weeks) and ‘maternal’ late-onset (≥34 weeks) phenotypes (Magee et al., 2022). More recently, a large-scale prospective cohort study (n = 9,102) leveraging mid-trimester maternal cell-free RNA transcriptomics has unveiled two distinct molecular endotypes. One is placenta-related type, characterized by the overexpression of placenta-specific genes, including PAPPA2. This form is closely associated with syncytiotrophoblast stress and placentation defects, clinically correlating predominantly with severe early-onset PE. Conversely, the second endotype is immune-related type, marked by elevated immune-cell gene signatures, such as CD163. This subtype reflects an exaggerated maternal systemic inflammatory response to physiological placental senescence or mild stress, clinically manifesting primarily as late-onset or mild PE (Redman et al., 2022; Elovitz et al., 2025).

This molecular stratification provides a novel framework for pharmacological prophylaxis. Precision treatment becomes the standard. Aspirin and low molecular weight heparin are the agents that enhance placental perfusion for the placental endotype. Besides, the immune and maternal endotypes require a strategic pivot. Therapeutic strategies should focus on metabolic or immune modulation. Metformin and statins target specific pathophysiology to ensure maximum efficacy (Ives et al., 2020; Dimitriadis et al., 2023; Elovitz et al., 2025).

## 3 Stratified aspirin prophylaxis: precision in preeclampsia high-risk subgroups

LDA remains the cornerstone of pharmacological prophylaxis for PE, however, its clinical efficacy exhibits heterogeneity in different high-risk subgroups (ACOG, 2020; Chappell et al., 2021; Dimitriadis et al., 2023). This variability suggests that it is necessary to develop individualized preventive strategies specifically targeting refractory subgroups complicated by multiple gestations, chronic hypertension, and metabolic disorders. Table 1 provides a comprehensive summary delineating the pharmacological mechanisms, targeted patient subgroups, and clinical efficacy profiles across specific phenotypes.

**TABLE 1:** Summary of mechanism-based prophylactic medications for preeclampsia across specific clinical phenotypes.

Medication	Target phenotype	Pharmacological mechanisms	Clinical efficacy and key effects	Key references
Standard LDA	Broad high-risk pregnancies	Inhibits COX-1 and suppresses TXA2 biosynthesis	Serves as the prophylactic cornerstone but exhibits highly heterogeneous efficacy across different subgroups	ACOG (2020); Chappell et al. (2021)
Escalated-dose aspirin (≥150 mg/d)	Multiple pregnancies	Provides dual COX-1 and COX-2 inhibition and neutralizes pathogenic EVs	Exerts profound anti-inflammatory benefits and overcomes COX saturation in augmented placentas	Zorzato et al. (2025); Rana et al. (2019)
LMWH alone or combined with LDA	Chronic hypertension with or without APS	Inhibits heparinase to protect endothelial glycocalyx, upregulates PlGF, and suppresses sFlt-1	Monotherapy reduces PE in specific cohorts, while early combination therapy reduces severe PE by approximately 50% in refractory phenotypes	Feng et al. (2023); Long et al. (2023)
Pravastatin	Metabolic high-risk populations	Activates the HO-1/CO pathway and inhibits chemerin expression	Timing-dependent: Early initiation (≤20 weeks) reduces PE risk by 61% with a favorable maternal-fetal safety profile	Mészáros et al. (2023); Khalili et al. (2025)
Metformin	Obese non-diabetic versus diabetic pregnancies	Suppresses sFlt-1 and sEng, and improves endothelial and mitochondrial function via the HIF-1α pathway	Phenotype-dependent: Significantly reduces PE in obese non-diabetic cohorts but lacks vascular protective effects in diabetic pregnancies	Syngelaki et al. (2016); Patel et al. (2024)

PE, preeclampsia; LDA, Low-dose aspirin; COX, cyclooxygenase; TXA2, Thromboxane A2; EVs, Extracellular vesicles; LMWH, Low-molecular-weight heparin; APS, antiphospholipid syndrome; PlGF, placental growth factor; sFlt-1, Soluble fms-like tyrosine kinase-1; sEng, Soluble endoglin; HO-1, Heme oxygenase-1; CO, carbon monoxide; HIF-1α, Hypoxia-inducible factor-1, alpha.

### 3.1 Refining prophylaxis in multiple gestations: targeting the supraphysiological vesicle burden

Multiple gestations confer an approximately threefold increase in the risk of PE (Chappell et al., 2021). However, prophylactic strategies for this population have long been challenged by a critical mismatch exists between standard dose and clinical efficacy. Observational data showed that conventional LDA regimens (75–100 mg/d) frequently exhibited limited efficacy in preventing PE in multiple gestations (Zhou et al., 2023). In contrast, evidence from multiple studies and systematic reviews have demonstrated that when initiated in the first trimester (<16 weeks) and maintained with high adherence (>90%), higher LDA dosages (≥100 mg/d, particularly 150–160 mg/d) can significantly reduce PE risk by 55%, highlighting a distinct dose-response relationship (Ye et al., 2021; Kalafat et al., 2020; Zorzato et al., 2025; D'Antonio et al., 2023).

The necessity for escalated dosages prompts a critical pharmacological thinking: why do twin pregnancies need to exceed the standard treatment threshold? Conventional hypotheses attribute this increased dosage requirement to the expanded placental mass and the concomitant surge in anti-angiogenic markers, such as sFlt-1. In this context, achieving complete and sustained acetylation of platelet cyclooxygenase-1 (COX-1) likely demands higher aspirin concentrations to sufficiently suppress thromboxane A2 biosynthesis and improve placental perfusion.

Furthermore, while standard LDA selectively targets COX-1, higher prophylactic doses (150–160 mg/d) extend pharmacological inhibition to COX-2 (Zorzato et al., 2025). This concurrent COX-2 blockade confers significant systemic anti-inflammatory benefits, effectively mitigating the exaggerated inflammatory cascades and severe endothelial dysfunction driven by the increased placental mass in multiple gestations (Rana et al., 2019; Chappell et al., 2021). Therefore, these higher-dose regimens achieve a dual suppression of both COX-1-mediated thrombosis and COX-2-driven inflammation.

However, this enzyme saturation hypothesis alone may not fully cover the underlying pathology, pointing to the emerging regulatory role of extracellular vesicles (EVs) as a more comprehensive pathophysiological explanation. EVs function as nano- to micro-scale vectors of intercellular communication, actively secreted by cells to transport specific bioactive molecules. In preclinical and *in vitro* models, hypoxic trophoblasts release a profusion of dysfunctional extracellular vesicles under the pathological condition of PE, acting as pathogenic messengers that induce widespread platelet aggregation and inflammasome activation (D'Crus et al., 2025; Kothandan et al., 2025). In multiple pregnancies, characterized by increased placental mass, may produce a supraphysiological load of pathogenic EVs, leading to systemic inflammatory cascades that is more resistant to containment. Furthermore, basic laboratory research has confirmed that aspirin could block this EVs-mediated platelet activation, mitigating subsequent trophoblast injury.

Therefore, administering dosages of 100 mg/d or higher, particularly in the 150–160 mg/d range (Poon et al., 2019; Ives et al., 2020; Magee et al., 2022), may have a dual therapeutic imperative in multiple gestations. On one hand, this regimen serves to overcome COX-1 enzyme saturation as understood in classical pharmacology. On the other hand, extrapolating from preclinical evidence, it may concomitantly neutralize the pathological signaling transmitted by an excess of pathogenic EVs. This synergistic mechanism between improving placental hemodynamics and inhibiting EVs-mediated signaling constitutes the theoretical foundation for the prophylactic efficacy of high-dose aspirin in this specific high-risk population. To validate this composite hypothesis *in vitro*, further in-depth pharmacodynamic and mechanistic studies are warranted.

### 3.2 Prophylaxis in superimposed preeclampsia: targeting endothelial dysfunction with aspirin and LMWH

Although chronic hypertension is a major risk factor associated with a fivefold increase in PE, the prophylactic efficacy of LDA monotherapy in this high-risk population has yielded inconsistent results. A recent cohort study demonstrated that prophylaxis initiated prior to 16 weeks of gestation failed to reduce the risk of superimposed PE in such pregnant women (Derrah et al., 2024). This therapeutic limitation may be related to the complexity of pathological mechanisms of PE caused by chronic hypertension, which involves profound endothelial injury and oxidative stress. While LDA as a selective COX-1 inhibitor may effectively suppress thromboxane A2 biosynthesis, it cannot reserve the damaged vascular endothelial barrier (Asiimwe et al., 2025).

Combined administration of LMWH and LDA shows a unique therapeutic value in this clinical context. Mechanistically, LMWH has been proposed for PE prophylaxis due to its pleiotropic properties that transcend conventional anticoagulative effects to preserve endothelial integrity. In preclinical models, LMWH inhibits heparinase, thereby preventing the degradation of endothelial heparan sulfate proteoglycans and maintaining vascular barrier integrity to attenuate leakage (Feng et al., 2023). Concurrently, animal and cellular studies suggest that LMWH may upregulate PlGF and suppress sFlt-1 release to directly optimize the microenvironment for extravillous trophoblast invasion (Baroutis et al., 2025).

Transitioning from these preclinical hypotheses to human clinical applications, evidence indicates that while LMWH monotherapy demonstrates a reduction in PE incidence among specific high-risk cohorts without established thrombophilia (Cruz-Lemini et al., 2022; Chen et al., 2024), its prophylactic superiority fundamentally relies on both combination regimens and strict intervention timing. Individual patient data meta-analyses reveal a clear temporal dependency: pathological reversal demands intervention during early placental remodeling, specifically prior to 16 weeks of gestation. Therapeutic efficacy is significantly compromised if treatment is delayed beyond this critical window (Rodger et al., 2016; Cruz-Lemini et al., 2022). Furthermore, the success of this strategy is highly phenotype-specific. Human clinical investigations confirm that early combination therapy of LDA and LMWH substantially improves maternal and perinatal outcomes, reducing the incidence of severe PE by approximately 50% in highly refractory phenotypes, such as patients with chronic hypertension complicated by positive antiphospholipid antibodies (Long et al., 2023). Ultimately, this synergistic dual-pathway strategy through the antithrombotic effects of LDA and the endothelial protection of LMWH theoretically overcomes the limitations of monotherapy.

Preventive strategy for chronic hypertension is evolving from single-agent antiplatelet therapy toward multi-target interventions based on pathophysiological classification. For example, the rational combination of antiplatelet agents and anticoagulants aims to overcome the efficacy limitations of current monotherapies by applying mechanistic complementarity.

### 3.3 Precision therapy for metabolic dysregulation: evaluating the efficacy of statins and metformin

Metabolic dysregulation fundamentally compromises pregnancy outcomes. Specifically, hyperglycemia and obesity induce oxidative stress and activate p38/MAPK signaling, thereby impairing extravillous trophoblast invasion and increasing the risk of PE. Pravastatin offers a novel solution. However, the clinical perspective on statin use during pregnancy is undergoing a paradigm shift, evolving from an absolute contraindication to a potential therapeutic intervention. Historically, these agents were contraindicated due to theoretical concerns that the impairment of fetal cholesterol synthesis could lead to teratogenicity. Nevertheless, recent evidence demonstrates that pravastatin crosses the placental barrier only minimally due to its distinct hydrophilic properties. Furthermore, multiple clinical cohort studies have indicated that *in utero* exposure to pravastatin is not associated with an increased risk of congenital malformations (Costantine et al., 2021).

Regarding preclinical and *in vitro* evidence, recent laboratory models have provided a strong mechanistic rationale for the application of pravastatin. *In vitro* studies confirmed the capacity of pravastatin to reverse high-glucose-induced cytotrophoblast dysfunction. Pantho et al. demonstrated that the agent reversed the downregulation of VEGF and PlGF, while concurrently suppressing the upregulation of sFlt-1 and sEng. This modulation restored the balance between urokinase-type plasminogen activator and its inhibitor to improve cytotrophoblast cell migration ability (Pantho et al., 2024). Furthermore, studies utilizing animal and cellular models suggest that its protective mechanism was related to the activation of the heme oxygenase-1/carbon monoxide pathway, which systemically ameliorates endothelial function and augments nitric oxide bioavailability (Aldika Akbar et al., 2025). Notably, a novel metabolic-vascular target was identified by Tan et al. (2024) in *Hypertension*. It revealed that pravastatin could inhibit the expression and release of placental chemerin, a vasoconstrictive adipokine known to be elevated in PE and associated with adverse pregnancy outcomes. Pravastatin exerts a dual effect. It not only reduces chemerin and sFlt-1 secretion, thereby increasing free PlGF levels, but also antagonizes chemerin-mediated vasoconstriction and inflammation by upregulating nitric oxide synthesis and low-density lipoprotein receptor expression (Pantho et al., 2024). This provides a new molecular basis for deploying pravastatin in the prophylaxis of metabolic-related PE.

Although these mechanistic advantages have begun to translate into clinical benefits, their prophylactic efficacy remains highly dependent on the timing of intervention. Recent comprehensive meta-analyses of human clinical studies have demonstrated that early pravastatin initiation (≤20 weeks of gestation) is associated with a significant reduction in the risk of PE and preterm birth among high-risk pregnancies (Mészáros et al., 2023; Akbar and Dekker, 2024; Khalili et al., 2025). However, interpreting these favorable outcomes requires caution due to the considerable heterogeneity in trial designs, particularly regarding dosing regimens, timing of enrollment, and the specific criteria for high-risk populations across pivotal studies (Provinciatto and Barbalho, 2025). Notably, despite historical contraindications, recent systematic reviews substantiate the favorable maternal-fetal safety profile of pravastatin, indicating an absence of significant adverse effects alongside consistent improvements in neonatal outcomes (Gera et al., 2025; Khalili et al., 2025).

While the clinical success of pravastatin is primarily dictated by the precise timing of intervention, investigations into other metabolic modulators underscore the patient phenotype as another critical dimension of precision prophylaxis. For instance, preclinical evidence from *in vitro* and animal studies indicates that metformin exhibits multi-target pharmacological potential in preventing PE. Its underlying mechanisms include the suppression of placental anti-angiogenic factors such as sFlt-1 and sEng, improvement of endothelial dysfunction, and regulation of mitochondrial function alongside the hypoxia-inducible factor-1alpha pathway (Tong et al., 2022; McEvoy et al., 2025). However, when these preclinical hypotheses are evaluated in human populations, clinical efficacy of metformin reveals significant population heterogeneity. In obese non-diabetic cohorts, a randomized controlled trial targeting pregnant women with a body mass index >35 kg/m^2^ demonstrated that 3,000 mg/d of metformin significantly reduced PE incidence from 11.3% to 3.0% (Syngelaki et al., 2016). A recent meta-analysis comprising 35 studies indicated that the administration of metformin resulted in a reduction in the risk of PE and gestational weight gain when compared to a placebo (Tarry-Adkins et al., 2021). In contrast, large randomized controlled trials involving diabetic pregnancies, such as the MiTy and the MOMPOD trials, failed to identify a significant effect of metformin on preventing PE (Feig et al., 2020; Patel et al., 2024). Furthermore, the MOMPOD trial, a high quality multicenter randomized controlled trial revealed that the addition of metformin to insulin therapy did not mitigate the risk of preterm PE. Moreover, metformin monotherapy was insufficient to change serum marker levels associated with PE and cardiovascular risk, including sFlt-1, PlGF, and VEGF, suggesting an absence of the anticipated vascular protective effects in this specific high-risk population (Patel et al., 2024).

In summary, prevention strategies are shifting toward personalization for high metabolic risk populations. While metformin maintains potential utility as an adjunctive prophylactic agent for primary maternal obesity via metabolic modulation, pregnant women who exhibit overt angiogenic imbalance or concomitant diabetes-related vascular risk warrant early intervention with pravastatin monotherapy or combined aspirin-statin therapy. These approaches represent the future of precision prophylaxis by relying on their regulation effect on the heme oxygenase-1/carbon monoxide pathway and adipokine Chemerin specific targets.

## 4 Conclusion and future perspectives

Strategies for PE prevention are currently undergoing a transition period from empirical and uniform protocols toward mechanism-based precision medicine. This review underscores that addressing the significant clinical heterogeneity of the syndrome requires therapeutic strategies matched to specific biological drivers, rather than relying on a single standard plan. Current evidence advocates for a critical transition toward targeted interventions: augmenting anti-inflammatory pathways in high-risk multiple gestations, repairing endothelial barrier integrity in hypertensive disorders of pregnancy, and correcting vascular metabolic imbalance in metabolic disorders. Ultimately, this field should evolve beyond traditional clinical risk stratification toward biomarker-driven randomized controlled trials. Future investigations should focus on biomarker-driven clinical trial designs that incorporate pre-randomization stratification based on transcriptomic signatures, such as placental PAPPA2 and maternal CD163 expression profiles. Only by integrating molecular diagnostics with targeted pharmacotherapy can clinical practice effectively translate pathological mechanisms into individualized clinical prophylaxis.
